# Investigation of Flat Clinching Process Combined with Material Forming Technology for Aluminum Alloy

**DOI:** 10.3390/ma10121433

**Published:** 2017-12-15

**Authors:** Chao Chen, Shengdun Zhao, Xiaolan Han, Yongfei Wang, Xuzhe Zhao

**Affiliations:** 1 School of Mechanical Engineering, Xi’an Jiaotong University, Xi’an 710049, China; yongfeio@126.com; 2 College of Mechanical and Electronic Engineering, China University of Petroleum (East China), Qingdao 266580, China; 3 Graduate School of Technology, Industrial and Social Sciences, Tokushima University, Tokushima 770-8506, Japan; 4 Mechanical Engineering College, Xi’an Shiyou University, Xi’an 710049, China; 5 School of Engineering Technology, Purdue University, West Lafayette, IN 47906, USA; longlong123sci@163.com

**Keywords:** flat-clinching, aluminum alloy, material flow, joining reliability

## Abstract

In recent years, the use of aluminum alloy has tended to increase for building lightweight automobiles to reduce their automotive weight, which is helpful to save energy and protect the environment. In order to join aluminum alloy, a flat-clinching process combined with material forming technology was investigated to join aluminum alloy sheets using an experimental and a numerical method. Al1060 was chosen as the material of the sheet, and DEFORM-2D software was used to build the numerical model. After the numerical model was validated by the experimental results, the influences of punch diameter and holder force on the materials deforming behavior of the clinched joint were analyzed using the numerical model. Then, the material flow, joining ability, and joining quality were investigated to assess the clinched joint. The results showed that an increase in punch diameter could give rise to an increase in neck thickness and interlocking length, while an increase in blank holder force induced a decrease in interlocking length and an increase in neck thickness. The joining quality could be increased by increasing the forming force. It can be concluded that a clinched joint has better joining quality for joining light-weight sheets onto automotive structures.

## 1. Introduction

Events such as the increase in greenhouse gases and the oil crisis in the 1970s have become catalytic factors to a requirement for reducing energy consumption [1]. Car safety, environmental protection, and energy saving have become three major key factors in automobile design [2,3], which have been given great attention from automobile manufacturers in recent years. It is important to build lightweight automotive structures to reduce the consumption of oil [4,5].

To build lightweight structures of automobiles, the use of aluminum alloy has tended to increase with its high specific properties [6,7]. The joining of lightweight materials has become a hot research topic in recent years [8]. Conventional mechanical clinching is a cold joining technology for joining metal sheets by a localized plastic forming process [9]. An interlock between two or more sheets is formed by clinching dies, such as fixed dies and extensible dies [10].

Previous studies have investigated the process parameters of the conventional clinching process and the geometrical parameters of conventional clinching tools. Lambiase [11] investigated the conventional clinching process parameters using numerical and experimental methods. Extensible dies were taken as the clinching tools in his study. The results showed that the clinching force mainly depended on the final bottom thickness and punch diameters. Oudjene and Ben-Ayed [12] carried out a parametrical study on the conventional clinching of metal sheets with the help of the Taguchi method. The results showed that the Taguchi method was effective for optimizing the geometrical parameters of the clinching dies. Lee et al. [13] investigated the influence of joining process parameters on the mechanical characteristics of aluminum alloy and advanced high-strength steel using a mechanical clinching method. They concluded that the interlocking length and neck thickness can determine the joining strength. Lambiase and Di Ilio [14] compared a fixed dis with extensible dies under different clinching forces. A peeling test and a shear test were conducted to assess the joining quality of these different joints. The results showed that a clinched joint with extensible dies could get a higher strength than a clinched joint with fixed dies during the peeling tests. Mucha [15] investigated the effect of die depth, die groove shape, and die radius of clinching tools on the mechanical properties of a joint with advanced high-strength steel. It was determined that the die groove width had an important influence on the mechanical properties and material flow of the clinched joint.

The conventional clinching process is applied to join many materials, such as copper alloy [16], titanium alloy [17], aluminum alloy [18], ultra-high strength steel [19], galvanized steel [20], Carbon Fiber Reinforced Polymer [21], and Glass Fiber Reinforced Polymer [22]. Different materials have different mechanical properties and melting points, which makes the joining of different materials difficult using welding technology [23]. However, the conventional clinching process is effective and suitable for joining sheets with different materials. In Lambiase and Di Ilio’s paper [24], a polymer sheet was joined with an aluminum alloy sheet using a mechanical clinching method. Gao et al. [25] dealt with the mechanical clinching of an aluminum AA6111 sheet with galvanized SAE1004 steel. The clinched joint with different materials could get a high static strength. Lee et al. [26] carried out some clinching experiments to join dissimilar materials: aluminum alloy with hot-pressed 22MnB5 steel, Carbon Fiber Reinforced Polymer, and DP780 steel. The clinching process was proved to be feasible for joining dissimilar materials.

Many experimental studies have been conducted to investigate the conventional clinching process. These experiments usually consume too much time and too many resources, which make the experiments expensive and complicated. In order to solve these problems, finite element simulation is frequently used to investigate the conventional clinching process using different simulation software (DEFORM, ABAQUS, MARC, LS-DYNA, and ADINA). Lambiase and Di Ilio [27] developed a finite element model to analyze the material flow and evolution of contact force during the clinching process with extensible dies. The finite element model was validated by clinching experiments. Mesh distortions may be generated during the finite element simulation process, which may affect the accuracy of the numerical results. He et al. [28] also carried out experimental and numerical investigations on extensible die clinching. In order to save time and cost, Eshtayeh and Hrairi [29] used finite element simulation to carry out a multi-objective optimization on the geometrical shape of fixed joining tools with the Taguchi method. Finite element simulation was proved to be efficient to optimize the clinching tools and investigate the metal flow and deforming characteristics.

In the conventional clinching process, a punch is applied to compress two or more sheets, which may form an exterior protrusion on one side of the sheet. With the redundant protrusion, it is not suitable for a clinched joint to be applied in visible places. This has become one of the main problems affecting the wide use of a clinched joint. Chen et al. [30] proposed a two-step clinching process to obtain a clinched joint with a lower protrusion. A reshaping process was combined with a conventional clinching process in their study. A redundant rivet was required during the two-step process, which may increase the joining cost. Wen et al. [31] studied the flat hole joining process to form a joint with a flat surface. A hole must be pre-punched before the joining process, which could reduce the process’s working efficiency. Neugebauer et al. [32] gave an overview on the development of the die-less joining process. Although the height of a redundant protrusion was reduced, a redundant protrusion could not be avoided. Lüder et al. [33] investigated the effects of wood moisture content on a clinched joint with aluminum alloy and wood. The joint was formed with no redundant protrusion after the joining process. This flat-joining method should be investigated in depth, especially using both an experimental method and a numerical method. The main process parameters, deforming characteristics, material flow, joining ability, and joining quality should be investigated to promote the development of this joining technology.

In the present work, a flat-clinching process combined with material forming technology was investigated using an experimental and a numerical method. The numerical model was built using DEFORM-2D software, and Al1060 was chosen as the material of the sheet. The numerical model was validated by the experimental result, which proved that it was feasible to use the numerical model for predicting the outcome of a flat-clinching process with an accurate result. The influence of punch diameter and holder force on the materials deforming behavior of the clinched joint was analyzed using this numerical model. Then, the material flow, joining ability, and joining quality were investigated to assess the clinched joint. It is proved that a clinched joint has better joining quality for joining Al1060 sheets.

## 2. Finite Element Methods

### 2.1. Numerical Model

A two-dimensional axisymmetric numerical model was built in the finite element simulation software DEFORM-2D. Compared with a three-dimensional numerical model, the two-dimensional axisymmetric numerical model can save a lot of time because it uses a small number of elements.

**Figure 1:**
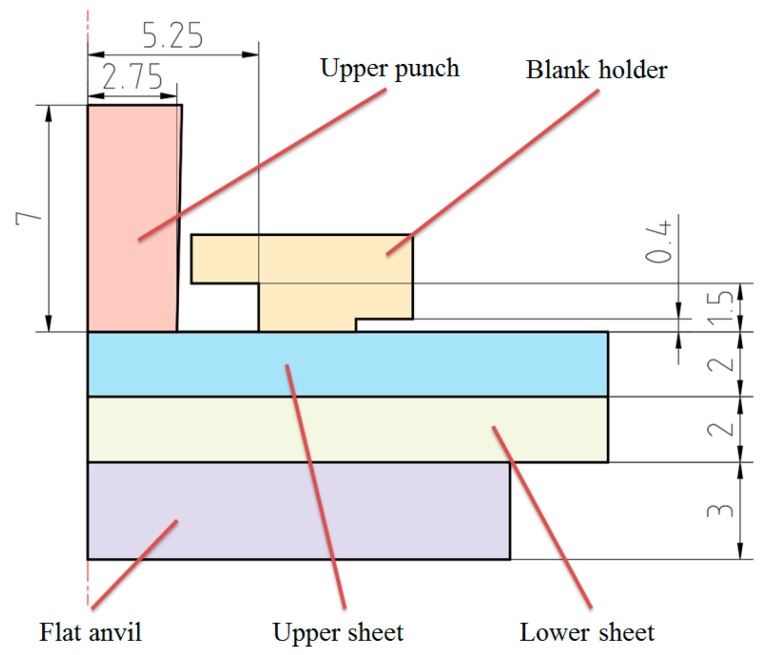
Main geometrical parameters of the numerical flat-clinching tool model.

In recent years, aluminum alloy has been widely applied to build mechanical structures because of its good forming abilities. Al1060 was taken as the sheet material in this finite element simulation. The thickness of the sheet was set to 2.0 mm. The Poisson’s ratio of the Al1060 is 0.33; its Young’s modulus is 54.5 GPa; and its tensile strength is 117.9 MPa. The sheet material was modeled as a plastic body, while the flat-clinching tools were modeled as rigid bodies. Quadrilateral elements were used to mesh the sheets, involving 3938 elements with 4140 nodes in the numerical model. The flat-clinching tools consist of an upper punch, a fixed flat anvil, and a blank holder. The main geometrical parameters of the numerical clinching tool model are shown in Figure 1.

The Coulomb friction law is suitable for a numerical simulation of the clinching process. Wen et al. [34], He et al. [28], Lambiase et al. [27], Oudjene et al. [12], Eshtayeh et al. [29], and many other researchers used the Coulomb friction law to simulate the clinching process. The numerical results agreed well with the experimental results. Thus, the Coulomb friction law is adopted in this study. Lambiase et al. [27] and He et al. [28] assumed the friction coefficient between the clinching tools and the sheets to be 0.15; Oudjene et al. [12] and Wen et al. [34] assumed the friction coefficient between the lower sheet and upper sheet to be 0.3. The numerical results using these friction coefficients agreed well with the experimental results. In this study, the Coulomb friction coefficient between the flat-clinching tools and the sheets was assumed to be 0.15, while the coefficient between the lower sheet and upper sheet was assumed to be 0.3. The automatic remeshing method was adopted to avoid large mesh distortion in the finite element simulation. The fixed flat anvil was fixed, and the upper punch was set to move downward with a forming speed of 0.4 mm/s.

### 2.2. Numerical Work Plan

**Figure 2:**
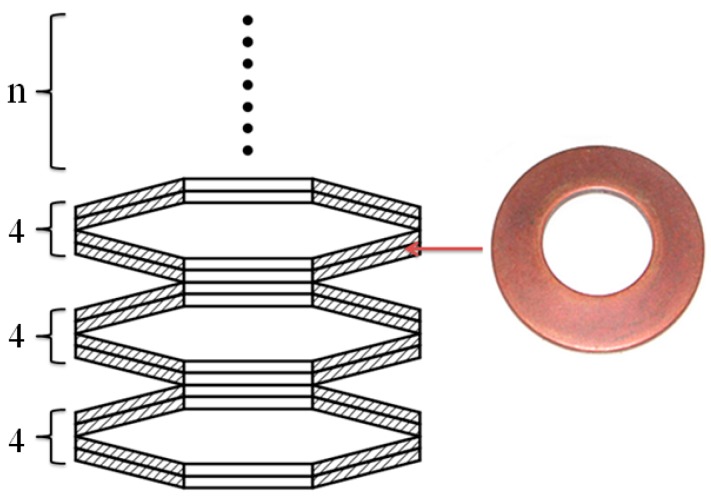
Disc spring used in this study.

**Figure 3:**
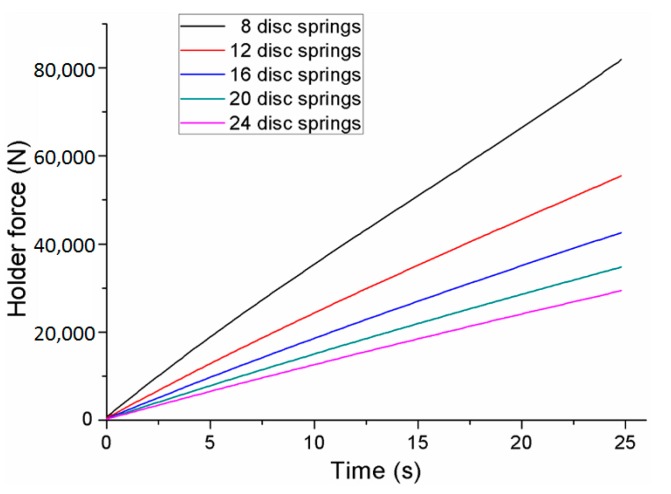
Holder forces generated by different combinations of disc springs.

The upper punch diameter, clinching force, and blank holder force have an important influence on the material flow, joining quality, and deforming characteristics of a clinched joint. The variation of punch diameters needs many different upper punches, which may increase the cost of the experimental study. So, the influence of upper punch diameters on the materials deforming behavior was investigated by finite element simulation. The different punch diameters of 4.3, 4.7, 5.1, 5.5, and 5.9 mm were adopted in the numerical simulation. The clinching force can be varied by controlling the clinching machine, which is easy to operate by an experimental method. The holder force applied to the blank holder was generated by various combinations of disc springs as shown in Figure 2. The holder force can be varied by varying the number of disc springs. A change in the disc springs may give rise to a change in the geometrical parameters of the clinching tools, which makes the experiments complicated. So, the influence of holder forces on the materials deforming behavior was also investigated by finite element simulation. The holder forces generated by 8, 12, 16, 20, and 24 disc springs are shown in Figure 3.

## 3. Experimental Procedures

### 3.1. Flat-Clinching Process

**Figure 4:**
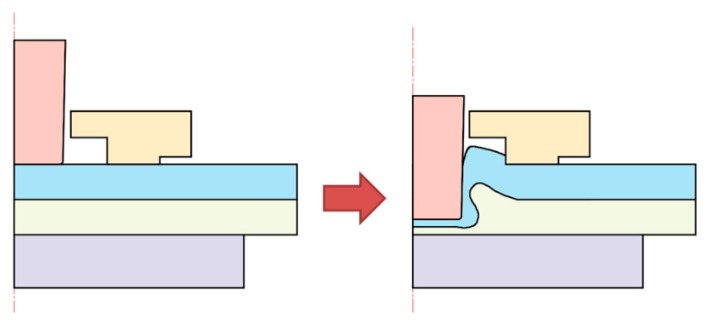
Flat-clinching process.

As shown in Figure 4, a mechanical joining interlock is formed with sheet deformation during the flat-clinching process. Two Al1060 sheets were piled onto a fixed flat anvil. The upper punch moved straight down to press the sheets with different forming forces. With the blank holder to restrict the sheets, the materials which flowed radially would turn to flow upward. Sheet materials which moved opposite to the punch’s moving direction would be clinched between the clinching tools. Thus, a joining interlock was formed, which clinched the sheets together.

**Figure 5:**
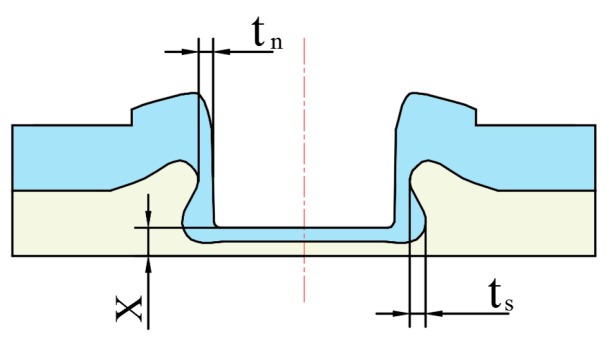
Main geometrical parameters of the clinched joint.

A mechanical structure is produced to clinch the sheets. Thus, the geometrical shape determines the joining ability and the quality of the flat-clinched joint. The shape’s main parameters, including the bottom thickness (*X*), interlocking length (*t_s_*), and neck thickness (*t_n_*), are shown in Figure 5.

**Figure 6:**
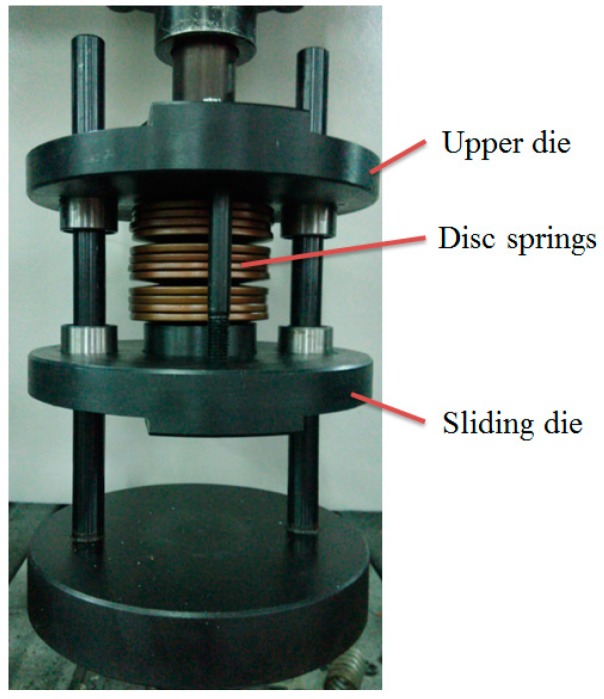
Flat-clinching tools.

The flat-clinching experiments were conducted on an Instron 5982 testing machine (Instron Company, Grove City, PA, USA). Joining dies made by molded steel are shown in Figure 6. The forming velocity was 0.4 mm/s during the joining process. The upper punch is installed on the upper die, and the blank holder is installed on the sliding die. The disc springs are installed between the upper die and the sliding die. Thus, with the movement of the punch, a holder force can be generated on the blank holder.

### 3.2. Specimen Preparation and Quality Evaluation

To assess the joining reliability of the joint, a tensile test (testing tensile strength) and a shearing test (testing shearing strength) are always carried out. Coppieters et al. [35] pointed out that the shearing strength is always higher than the tensile strength. Therefore, a tensile test was used to assess the joining reliability of the clinched joint in this paper [36]. An Instron 5982 testing machine was also used to test the joining reliability of this joint using the tensile test. The maximum tensile force tested by this machine was treated as the tensile strength. The tensile speed was 2 mm/min in the tests.

**Figure 7:**
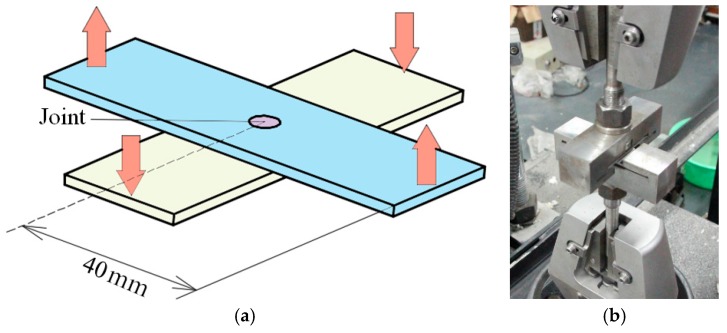
Specimen used for tensile test: (**a**) Specimen used for tensile test (**b**) tensile testing fixtures.

Figure 7a shows the specimen placed as cross-shaped type, which was prepared for tensile test. Figure 7b shows the tensile fixtures used in the tensile test. The lower fixture was used to fix the lower sheet, and the upper fixture was used to pull the upper sheet.

### 3.3. Experimental Work Plan

Forming force is one of the key parameters affecting the joining reliability of a clinched joint [37,38,39]. The forming force includes the holder force and the force on the punch during the flat-clinching process. Investigating the influence of the forming force is required. Different forming forces, namely 50, 60, 70, 80, and 90 kN, were used on the punch to form different joints. Then, the joining ability, joining quality, and failure modes of these different clinched joints were investigated and are discussed below. Five replications were performed for each condition.

## 4. Results and Discussion

### 4.1. Numerical Model Validation

Before using the numerical model to carry out the finite element simulation, a numerical model validation is reported to discuss the accuracy of the numerical model. The main geometrical parameters, such as neck thickness and interlocking length, affect the joining quality of a clinched joint [40]. Thus, a comparison of the numerical results and experimental data concerning the neck thickness and interlocking length are involved in the numerical model’s validation.

The experimental results of the neck thickness and interlocking length were measured by sectioning the clinched joints along the axial plane. Five repetitions for each joining condition were conducted to obtain the mean value.

**Figure 8:**
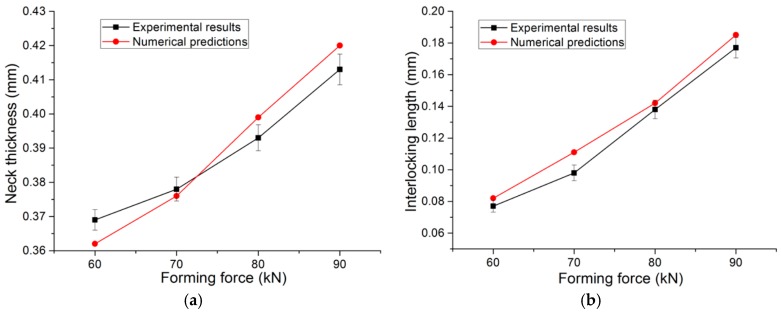
Comparison of the numerical predictions and experimental results: (**a**) neck thickness; (**b**) interlocking length.

**Figure 9:**
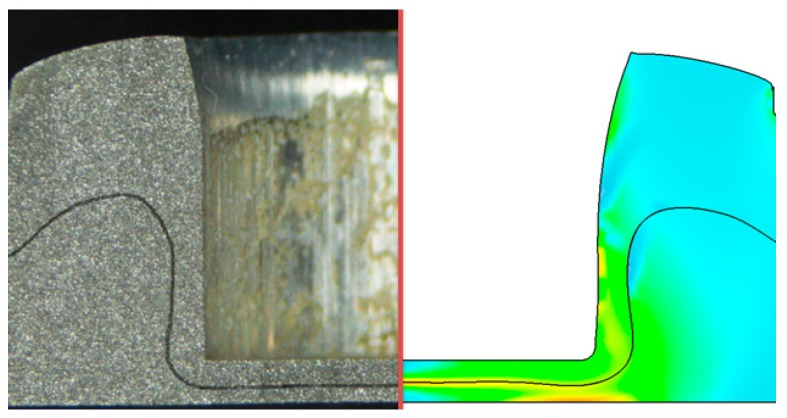
Numerical and experimental cross-sections of the clinched joint.

The comparison of the numerical results and experimental data is shown in Figure 8. The numerical results agreed well with the experimental data concerning the neck thickness and interlocking length of the clinched joint. The numerical and experimental cross-sections of the clinched joint are shown in Figure 9. The solid line and shade color are the results of the numerical simulation. The cross-section of the numerical model is similar to the experimental cross-section, which also proves that it is feasible to use the numerical model for predicting the outcome of a flat-clinching process with an accurate result.

### 4.2. Influence of Punch Diameter on the Materials Deforming Behavior

**Figure 10:**
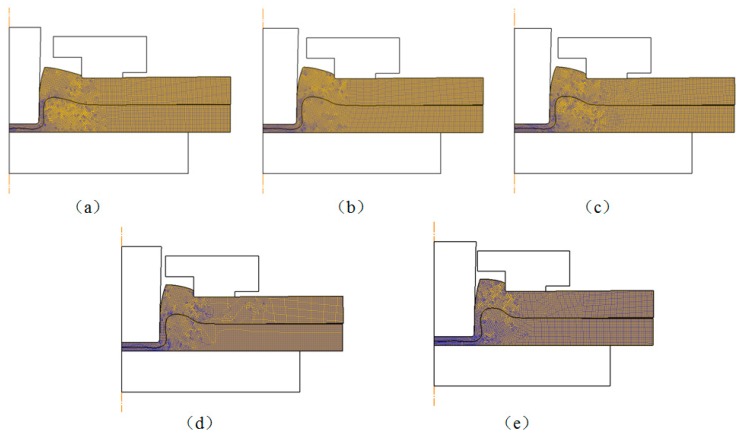
Shapes of the deformed zones resulting from the numerical simulations with different punch diameters: (**a**) punch diameter of 4.3 mm; (**b**) punch diameter of 4.7 mm; (**c**) punch diameter of 5.1 mm; (**d**) punch diameter of 5.5 mm; (**e**) punch diameter of 5.9 mm.

Punch diameter is one of the key parameters in the flat-clinching process. The influence of punch diameter on the materials deforming behavior is investigated in this section. The shapes of the deformed zones resulting from the numerical simulations with different punch diameters are shown in Figure 10. Different clinched joints were formed using different punch diameters. Other geometrical parameters of the clinching tools and the process parameters of the flat-clinching process were kept consistent.

**Figure 11:**
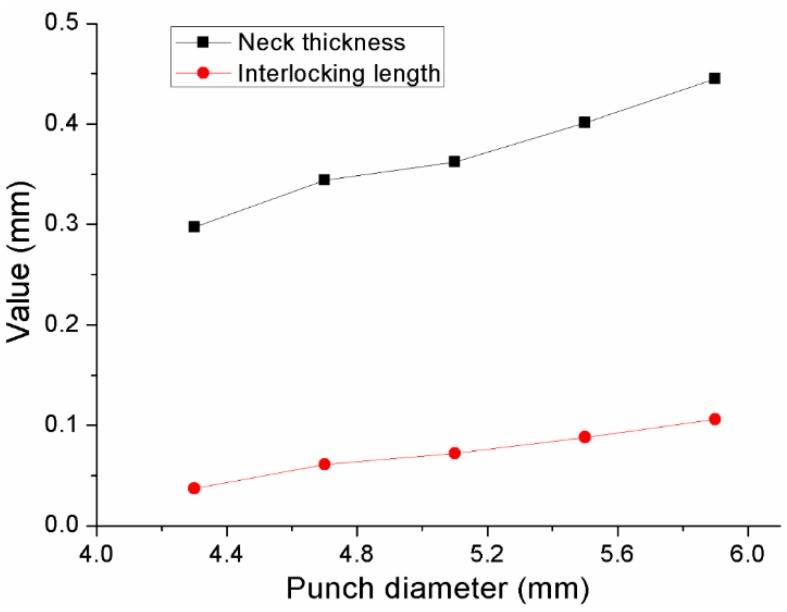
Neck thicknesses and interlocking length of the joints with different punch diameters.

The neck thicknesses and interlocking lengths of joints with different punch diameters are shown in Figure 11. It can be observed that an increase in punch diameter induces an increase in the neck thickness and interlocking length. In general, the joining quality follows a trend similar to that for the neck thickness and interlocking length. Therefore, an increase of the punch diameter can increase the joining reliability of the joint.

**Figure 12:**
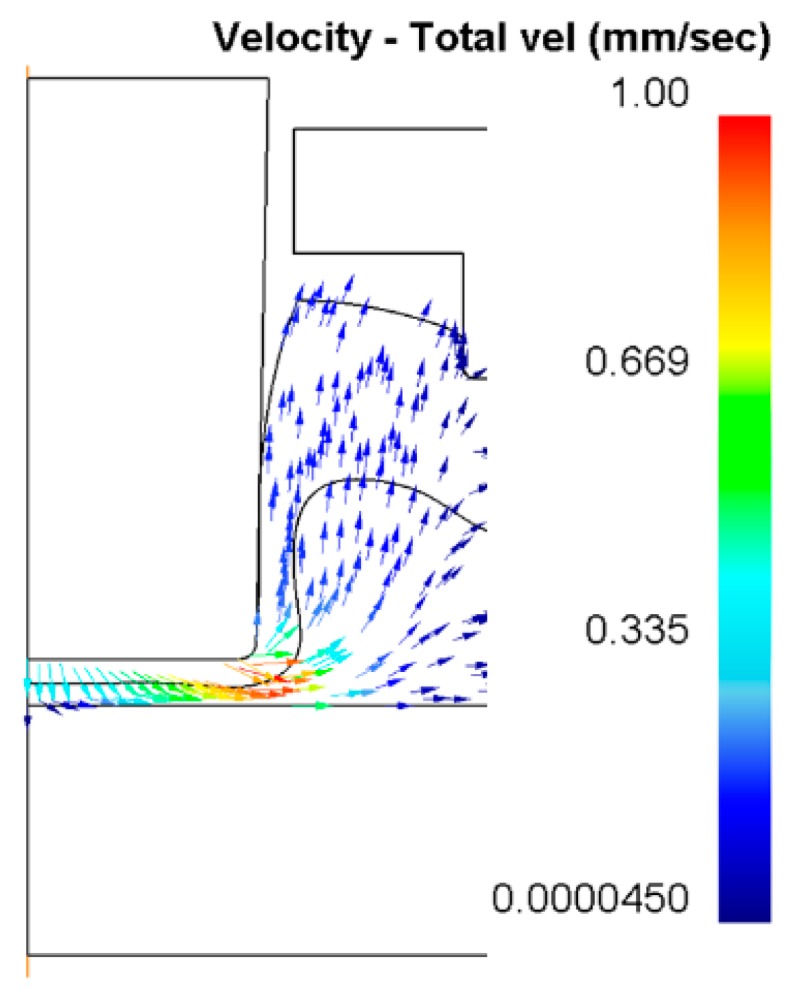
Velocity distribution of material at the bottom part using a punch diameter of 5.5 mm and a forming force of 90 kN.

As shown in Figure 12, materials of the bottom parts were pushed to flow down and radially with the downward movement of the upper punch, and materials around the neck were pushed to flow upward. The velocity reaches its highest value at the forming region at the bottom part under the upper punch. The materials were under severe plastic deformation, as shown in Figure 12, at this region. Those punches with a larger diameter pushed more materials of the bottom parts (higher material flow) to flow to the neck and interlock areas, which gave rise to the increase of the neck thickness and interlocking length. However, the joining area for joining the metal sheets is always limited in actual use, which also gives a limit to the use of the punch diameter. Within the limit of the actual operating conditions, it is better to use a punch with a large diameter in the flat-clinching process.

### 4.3. Influence of Holder Force on the Materials Deforming Behavior

**Figure 13:**
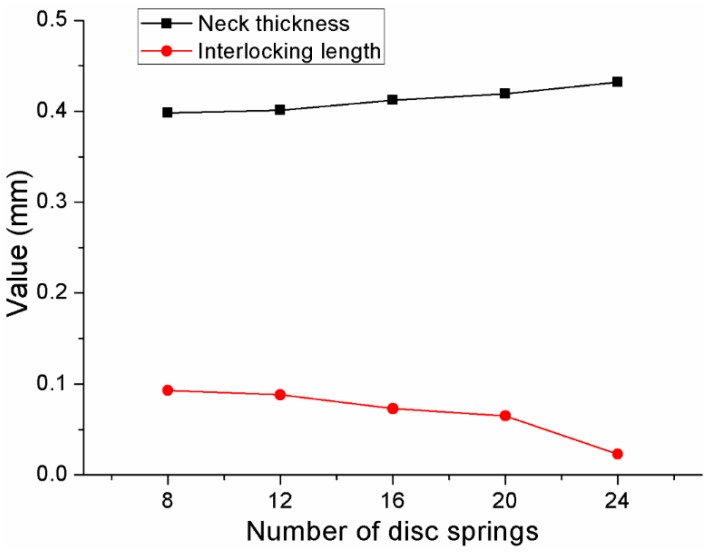
Neck thicknesses and interlocking lengths of the clinched joints with different combinations of disc springs.

Different clinched joints were formed using different blank holder forces. Other geometrical parameters of the clinching tools and the process parameters of the flat-clinching process were kept consistent. Different combinations of disc springs were used to generate different blank holder forces. The neck thicknesses and interlocking lengths of the clinched joints with different combinations of disc springs are shown in Figure 13. It can be observed that an increase in the blank holder force induces a decrease in the interlocking length and an increase in the neck thickness. With a smaller interlocking length, the joining reliability of the joint may be affected.

**Figure 14:**
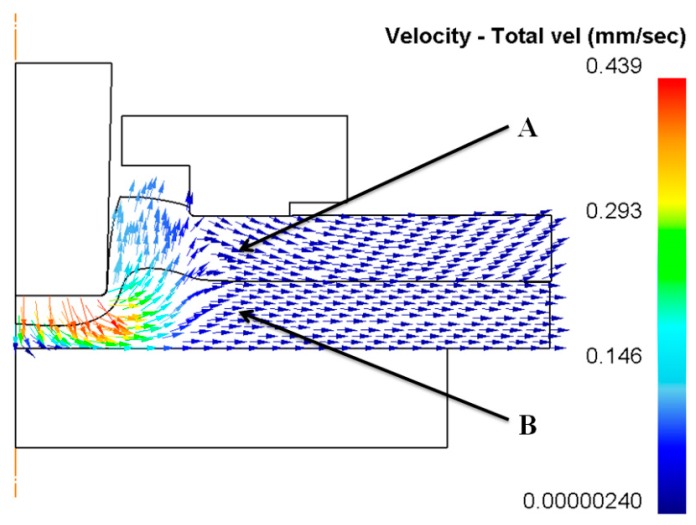
Material flow under the blank holder using a punch diameter of 5.5 mm and a forming force of 90 kN.

A die holder was applied to hold the Al1060 sheets on a flat anvil. As shown in Figure 14, the material of the sheets under the blank holder moved radially with the downward movement of the upper punch. The radial flow of the materials was restricted by the die holder in the flat-clinching process. Point ‘A’ and point ‘B’ on the upper sheet and lower sheet, respectively, were chosen to show the variation of velocity during the flat-clinching process.

**Figure 15:**
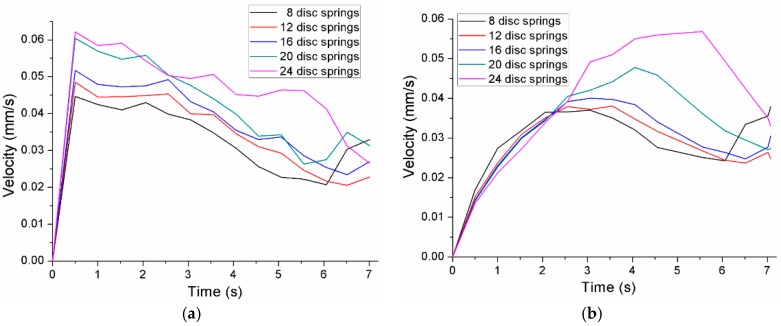
Velocities of point ‘A’ and point ‘B’ using a punch diameter of 5.5 mm and a forming force of 90 kN: (**a**) point ‘A’; (**b**) point ‘B’.

The velocities of point ‘A’ and point ‘B’ during each performance of the flat-clinching process with different combinations of disc springs are shown in Figure 15. The material under a lower holder force has a higher velocity than the material under a larger holder force. With a lower holder force, more material would flow in the radial direction without the restriction of the blank holder, which could generate a smaller interlocking length. In the flat-clinching process, a higher holder force is required to restrict the material flow.

**Figure 16:**
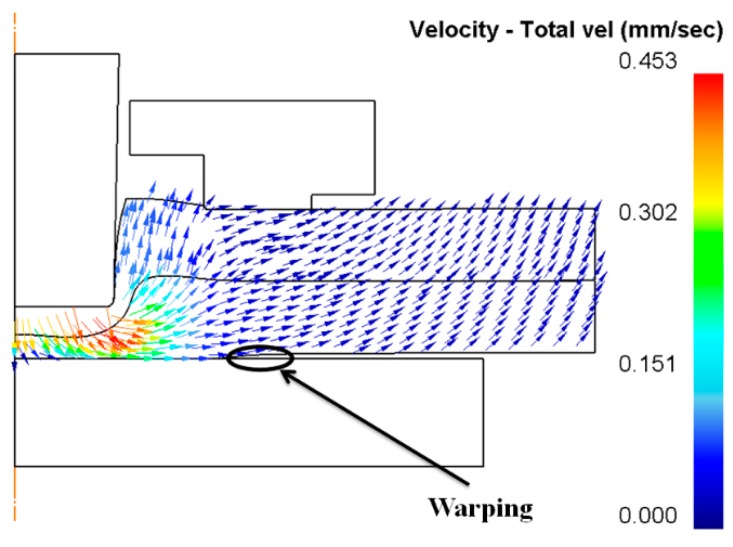
Warping of sheets under a lower holder force using a punch diameter of 5.5 mm and a forming force of 90 kN.

In addition, as shown in Figure 16, for the clinched joints under the blank holders with 20 disc springs and 24 disc springs, the sheets were warped during the clinching process. The holder forces were not enough to fix the sheets on the flat anvil, which could affect the joining quality and joining appearance of the clinched joints. The best way to solve this problem is to improve the blank holder force.

### 4.4. Analysis of Material Flow

**Figure 17:**
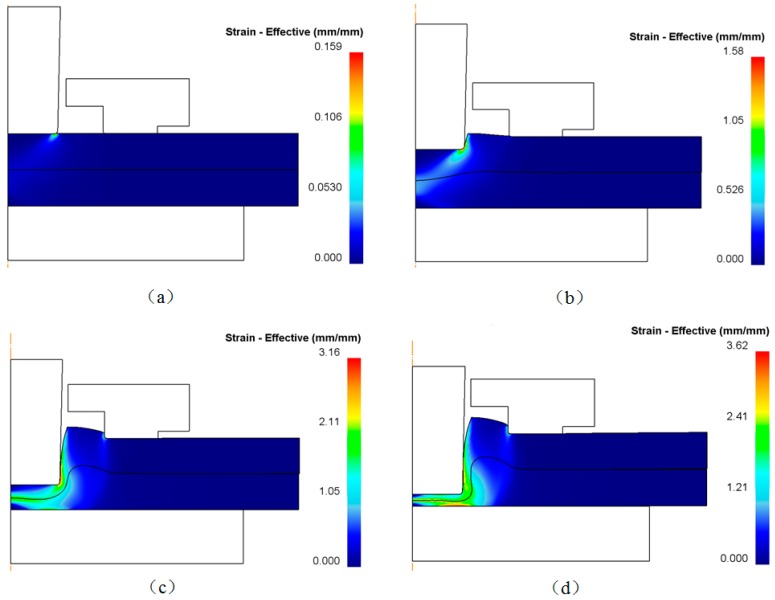
Material flow during the joining process: (**a**) local deforming; (**b**) drawing; (**c**) backward extruding; and (**d**) interlock forming.

The joining process consists of four stages as shown in Figure 17. The first stage is called local deforming; the second stage is called drawing; the third stage is called backward extruding; and the fourth stage is called mechanical interlock forming [41]. The evolution of metal flow determines the cross-sectional shape of the joint.

Firstly, the blank holder fixed two Al1060 sheets on the flat anvil. Then, a forming force was applied to the punch to compress the Al1060 sheets. The sheet materials would flow downward under the punch. With the limitation of the flat anvil, the material flowing downward would turn to flow radially. The die holder gave a limit to the radial material flow, which resulted in the upward flow of the sheet material. The upward material would be collected in the gap between the die holder and punch. During the third stage, the lower sheet material was also pushed to flow opposite to the punch direction. During the last stage, a mechanical joining interlock was formed, which clinched the two Al1060 sheets together. The flat anvil restricted the material flow downward, which resulted in a flat sheet surface with no exterior protrusion.

### 4.5. Joining Ability and Joining Quality

**Figure 18:**
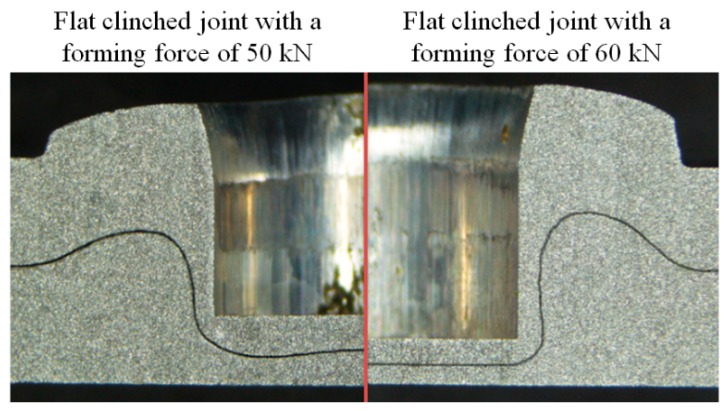
Comparison of the clinched joints with forming forces of 50 kN and 60 kN.

As shown in Figure 18, for the joint formed by a forming force of 50 kN, no mechanical joining interlock was produced, which meant that the sheets were not joined together. For the joints with joining forces of 60~90 kN, a mechanical joining interlock was produced, which meant that the sheets were joined together.

**Figure 19:**
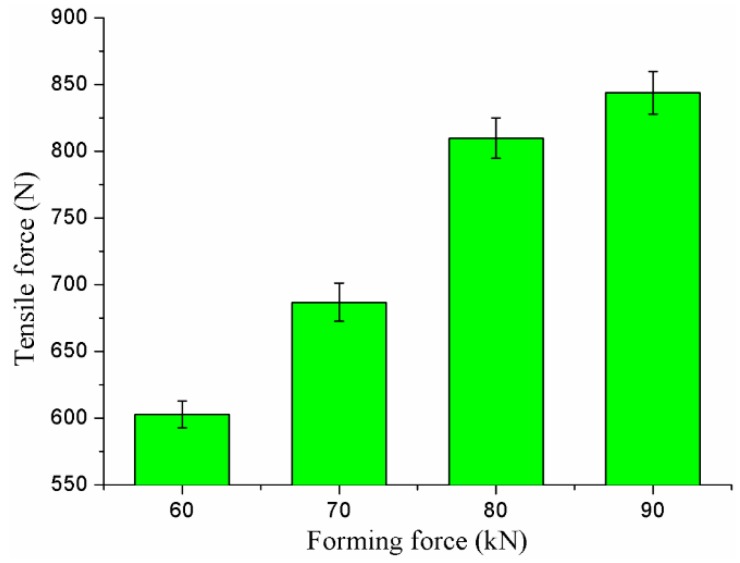
Tensile strengths of the clinched joints.

Tensile strength is always used to assess the joining reliability of the joint [42]. The static tensile strengths of the joints formed by joining forces of 60, 70, 80, and 90 kN are shown in Figure 19. Five shearing joints were used in the tests to obtain the average strength under each joining condition. The increased joining force also gave a rise to an increase of tensile strength. The tensile strength of the joint formed by a joining force of 60 kN was the lowest, while the tensile strength of the joint formed by a joining force of 90 kN was the highest.

**Figure 20:**
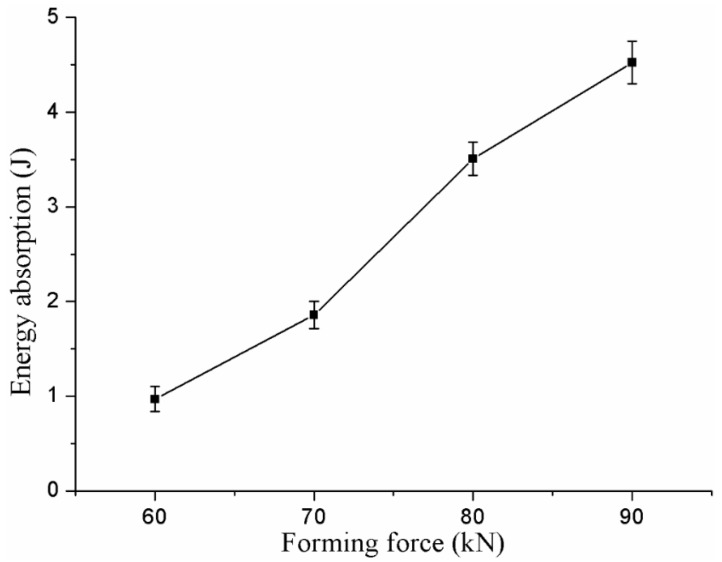
Energy absorption of the clinched joints.

Energy absorption in the process of failure is another important evaluation criterion for clinched joints [43]. A higher energy absorption in the failure process means a higher safety. The ability for energy absorption in the failure process is an essential parameter to evaluate the joining reliability. In order to protect the driver, it is required for the joint to have better performance for energy absorption in the automobile. The energy absorption of joints formed by the joining forces of 60, 70, 80, and 90 kN in the process of failure is shown in Figure 20.

The energy absorption of a joint was increased by increasing the joining force. The joint formed by a joining force of 90 kN had the highest energy absorption in the test, while the clinched joint formed by a joining force of 60 kN had the lowest energy absorption. The joint with a joining force of 90 kN had the longest displacement and highest strength in the tensile test, which gave rise to the highest energy absorption.

## 5. Conclusions

In the present work, a flat-clinching process combined with material forming technology was investigated using experimental and numerical methods. After the numerical model was validated with the experimental results, the influence of punch diameter and holder force on the materials deforming behavior of the clinched joint was investigated. Then, the material flow, joining ability, and joining quality were investigated to assess the clinched joint. The main conclusions of the present work can be drawn as follows:

(1) A punch with a larger diameter could push more materials of the bottom parts (higher material flow) to flow to the neck and interlock areas, which can give rise to an increase in the neck thickness and interlocking length. With a larger neck thickness and interlocking length, the joining quality of the clinched joint could be improved.
(2) An increase in blank holder force induces a decrease in interlocking length and an increase in neck thickness. With a lower holder force, more material would flow in the radial direction without the restriction of the blank holder, which could generate a smaller interlocking length. In the flat-clinching process, a higher holder force is required to restrict the material flow.
(3) A mechanical joining interlock is formed during the joining process. The material flow in the joining process consists of four stages: (a) local deforming; (b) drawing; (c) backward extruding; and (d) interlock forming.
(4) For the joints formed by a joining force of 50 kN, there is no mechanical interlock generated, while for the joints formed with the joining forces of 60, 70, 80, and 90 kN, a mechanical interlock was formed because of the plastic deformation. Joining reliability could be improved by increasing the forming force.
